# Supplementary figures and images for: JAK2-STAT Epigenetically Regulates Tolerized Genes in Monocytes in the First Encounter With Gram-Negative Bacterial Endotoxins in Sepsis
Source: Front Immunol. 2021 Nov 17;12:734652. doi: 10.3389/fimmu.2021.734652 (PMC8635809; doi:10.3389/fimmu.2021.734652)

**Figure S1**

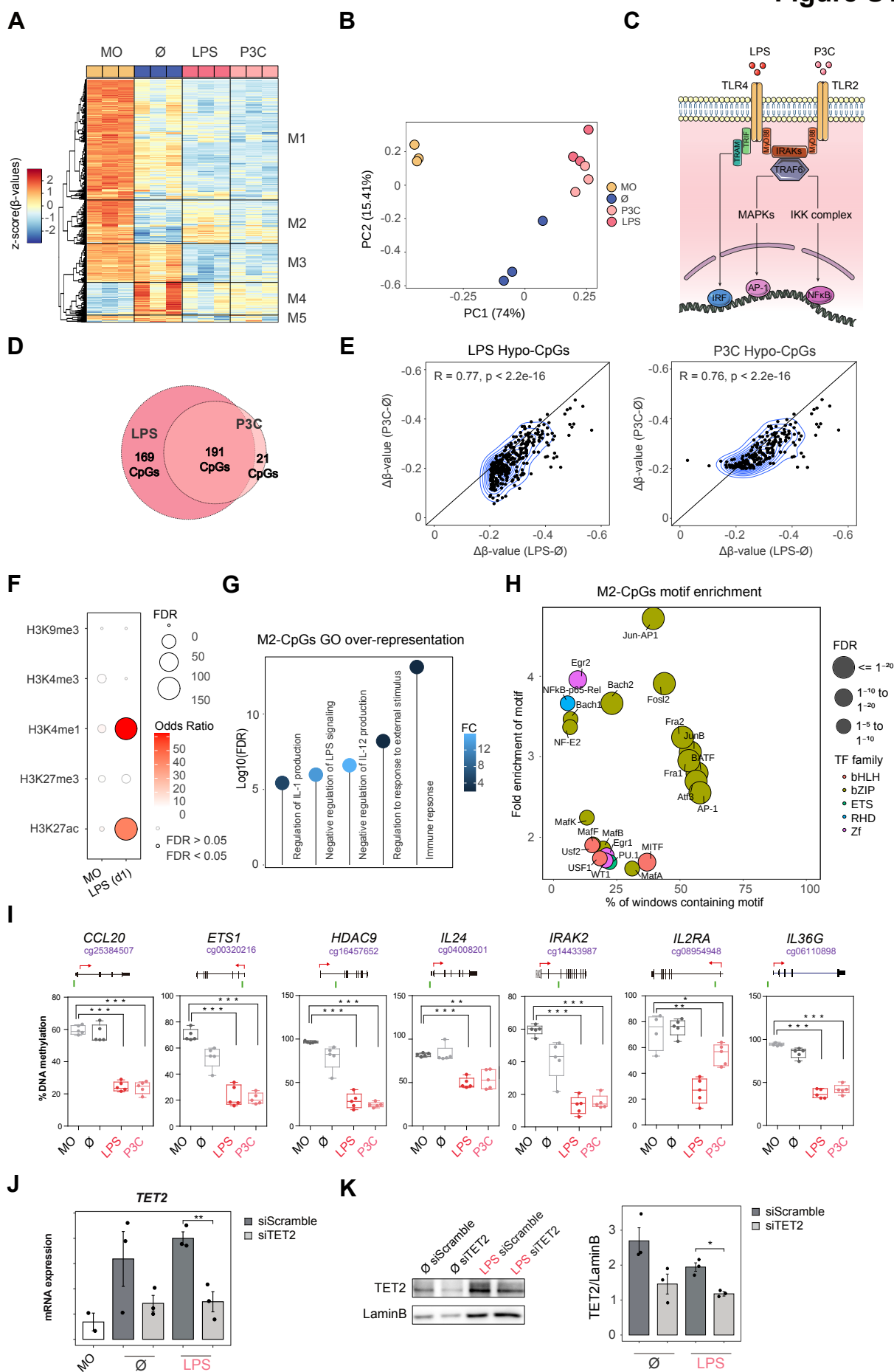

Supplement: Supplementary Figure S1 — DNA methylation profile of P3C-treated human CD14+ monocytes. (A) DNA methylation heatmap of differentially methylated CpGs obtained with all the possible contrasts between the represented groups (Δβ- ≥ 0.2, adjusted p (FDR) < 0.05). Scaled β-values are shown, ranging from -2 (lower DNA methylation levels, blue) to +2 (higher methylation levels, red). (B) Principal component analysis (PCA) of the CpGs shown in Supplementary Figure 1A. Principal components 1 and 2 are shown. (C) Simplified scheme of TLR4 and TLR2 signaling pathways. (D) Venn diagram of differentially methylated CpGs of LPS-treated versus untreated monocytes (LPS) and P3C-treated versus untreated monocytes (P3C). (E) Scatter plots of LPS hypomethylated and P3C hypomethylated CpGs showing differential of DNA methylation of LPS-treated versus untreated monocytes (x-axis) and P3C-treated versus untreated monocytes (y-axis). Contours in blue represent a 2D kernel density estimation. The R value and p values were obtained with Person’s correlation. (F) ChIP-seq data of depicted histone marks were downloaded from the Blueprint database. Over-representation of CpGs in histone marks were calculated using the Fisher’s test. Data points with FDR < 0.05 and Odds Ratio > 5 were considered significant. (G) GO (gene ontology) over-represented categories in M2 cluster CpGs. Fold change relative to background (EPIC array CpGs) and -log10(FDR) are shown. (H) Bubble scatterplot of TF binding motif enrichment for M2 cluster CpGs. The x-axis shows the percentage of windows containing the motif and the y-axis shows the magnitude of enrichment of the motif. Bubbles are colored according to the TF family. The FDR is indicated by the bubble size (selected TF with FDR ≤ 1e-05). (I) Percentage methylation level of some selected LPS-hypomethylated CpGs as obtained by bisulfite pyrosequencing in the LPS and P3C samples. (J) qRT-PCR analysis to validate the downregulation of TET2 by siRNA (siScramble used as control). Data are no [file DataSheet_1.pdf]

# Figure S2

**A**

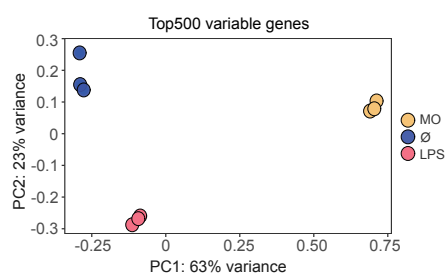

**B**

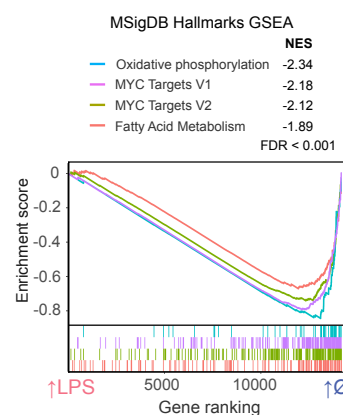

**C**

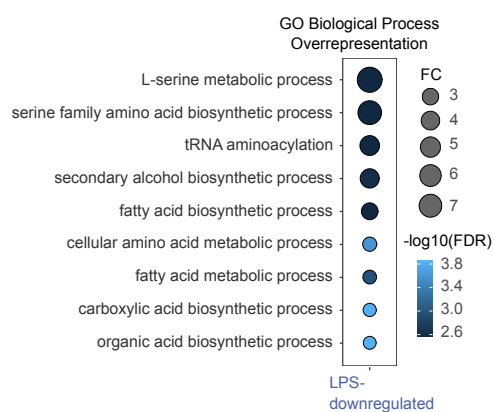

**D**

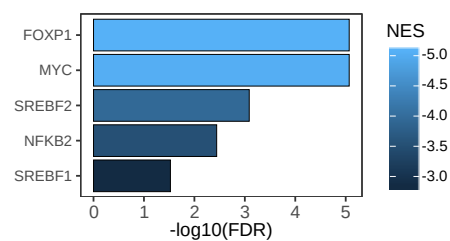

**E**

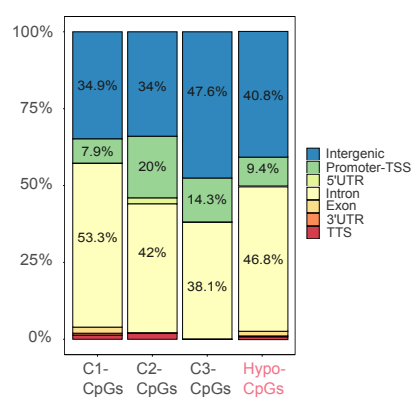

**F**

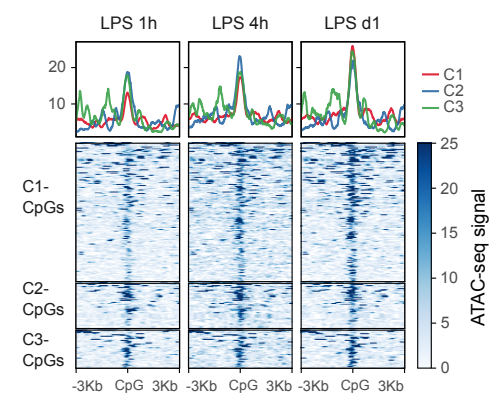

**G**

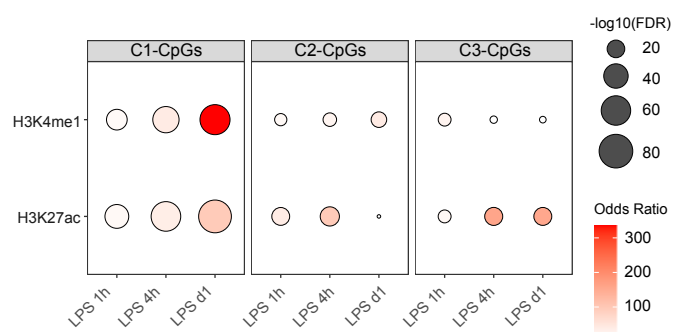

**H**

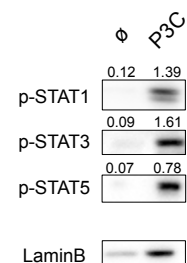

Supplement: Supplementary Figure S2 — Additional features of differentially expressed genes and associated CpGs. (A) Principal component analysis (PCA) of the 500 most variable genes. Principal components 1 and 2 are shown. (B) Gene set enrichment analysis (GSEA) of LPS-treated versus untreated (Ø) monocytes, using MSigDB hallmarks (H) as gene sets. Gene sets enriched in untreated (Ø) monocytes are shown. The running enrichment score and the normalized enrichment score (NES) are shown above the graph (FDR < 0.001). (C) Gene ontology (GO) over-representation of GO biological process categories. Fold change of LPS-downregulated genes relative to background and -log10(FDR) of the Fisher’s exact tests are shown. (D) Discriminant regulon expression analysis (DoRothEA) of LPS-treated versus untreated (Ø) monocytes. NES and -log10(FDR) of transcription factors (TF) enriched on the Ø side are shown (FDR < 0.05). (E) Barplot of genomic features as percentages of C1-, C2- and C3-associated CpGs (Figure 2H) compared with total hypo-methylated CpGs. (F) Accessibility (ATAC-seq) data of CpGs associated with C1 (in red), C2 (in blue) and C3 (in green) after 1, 4 and 24 hours of monocyte culture with LPS. Public ATAC-seq data were used [15] (G) ChIP-seq data of H3K27ac and H3K4me1 of LPS-treated monocytes were downloaded from the Blueprint database. Odds ratios were calculated with C1-, C2- and C3-associated CpGs, using EPIC array CpGs as background. Odds ratio and -log10(FDR) obtained from Fisher’s exact tests are shown. (H) Western blot of protein phosphorylated (p)-STAT1, p-STAT3 and p-STAT5 in P3C-treated relative to untreated (Ø) monocytes. LaminB was used as loading control. Signal of western blot bands was quantified and the pSTAT/LaminB ratios are shown above each protein. [file DataSheet_2.pdf]

Figure S3

A

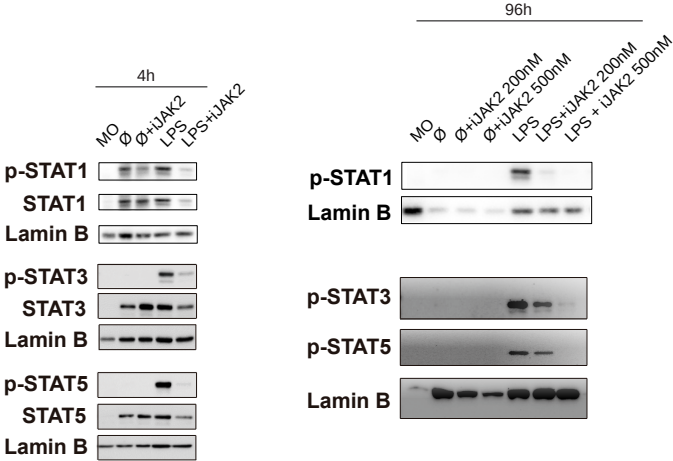

B

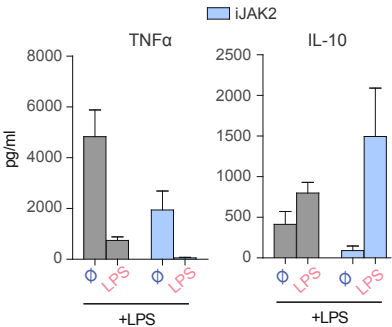

Supplement: Supplementary Figure S3 — Additional characterization of JAK2 inhibition in LPS-treated monocytes. (A) Western blot of JAK2-dependent STAT proteins in LPS-treated and untreated (Ø) monocytes, with and without JAK2 inhibitor (iJAK2) after 4 hours (left) or 96 hours (right) of treatment. LaminB was used as the loading control. (B) TNFα and IL-10 levels measured in cell supernatants to assess the tolerance state after JAK2 inhibition. [file DataSheet_3.pdf]

**Figure S4**

**A**

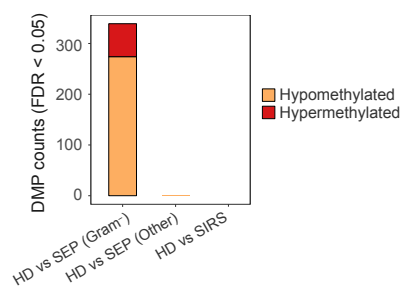

**B**

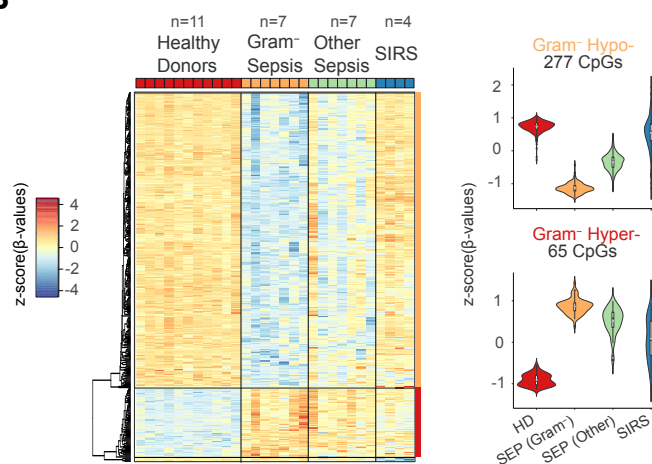

**C**

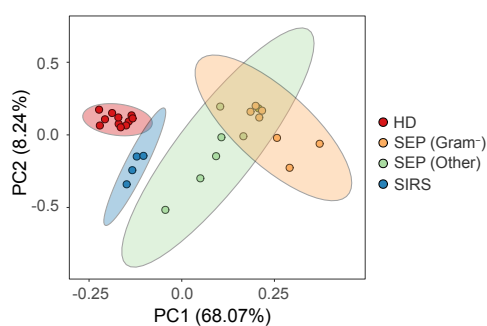

**D**

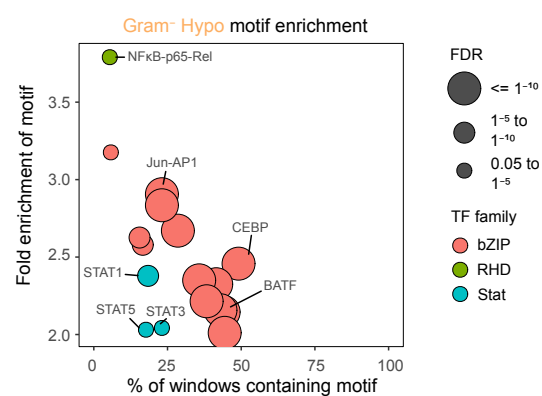

**E**

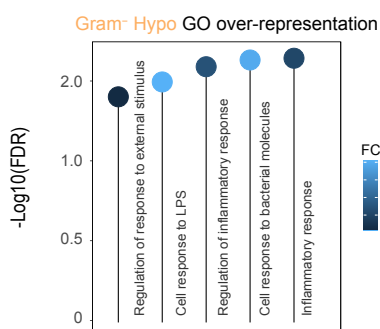

**F**

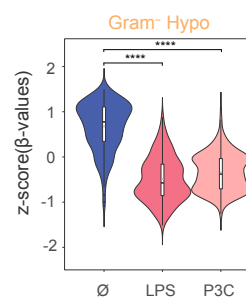

Supplement: Supplementary Figure S4 — DNA methylation alterations in CD14⁺ monocytes from Gram-negative sepsis. (A) Differentially methylated positions (DMPs) found in the comparisons of Gram⁻ sepsis (SEP), other sepsis and SIRS patients with healthy donors (HD) (FDR < 0.05). (B) DNA methylation heatmap of differentially methylated CpGs comparing all groups shown pairwise (FDR < 0.05). Scaled and batch-corrected β-values are shown, ranging from -4 (lower DNA methylation levels, blue) to +4 (higher methylation levels, red). On the right, violin plots of hypomethylated and hypermethylated clusters depicting normalized DNA methylation data. (C) Principal components 1 and 2 from a principal component analysis (PCA) of the DMPs from Supplementary Figure 4B are shown. (D) Bubble scatterplot of TF binding motif enrichment for Gram-negative hypomethylated CpGs. The x-axis shows the percentage of windows containing the motif; the y-axis shows the magnitude of enrichment of the motif. Bubbles are colored according to TF family. FDR is indicated by bubble size (selected TF with FDR ≤ 0.05). (E) GO (Gene Ontology) over-represented categories in the Gram-negative hypomethylated CpGs. The fold change relative to the background (EPIC array CpGs) and -log10(FDR) is shown. (F) Violin plot of normalized βvalues of the Gram-negative Hypomethylated CpGs in untreated monocytes (Ø), LPS-treated monocytes and P3C-treated monocytes (277 CpGs). [file DataSheet_4.pdf]
